# The effect of ZnO nanoparticle coating on the frictional resistance between orthodontic wires and ceramic brackets

**DOI:** 10.15171/joddd.2016.017

**Published:** 2016-06-15

**Authors:** Ahmad Behroozian, Mojgan Kachoei, Masumeh Khatamian, Baharak Divband

**Affiliations:** ^1^Assistant Professor, Department of Orthodontics, Faculty of Dentistry, Tabriz University of Medical Sciences, Tabriz, Iran; ^2^Associate Professor, Department Of Orthodontics, Faculty of Dentistry, Tabriz University of Medical Sciences, Tabriz, Iran; ^3^Professor, Inorganic Chemistry Department, Faculty of Chemistry, University of Tabriz, Tabriz, Iran; ^4^Researcher, Research Center for Pharmaceutical Nanotechnology, Tabriz University of Medical Sciences, Tabriz, Iran

**Keywords:** Friction, nanoparticles, orthodontics, porcelain, wire, ZnO

## Abstract

***Background.*** Any decrease in friction between orthodontic wire and bracket can accelerate tooth movement in the sliding technique and result in better control of anchorage. This study was carried out to evaluate frictional forces by coating orthodontic wires and porcelain brackets with zinc oxide nanoparticles (ZnO).

***Methods***. In this in vitro study, we evaluated a combination of 120 samples of 0.019×0.025 stainless steel (SS) orthodonticwires and 22 mil system edgewise porcelain brackets with and without spherical zinc oxide nanoparticles. Spherical ZnOnanoparticles were deposited on wires and brackets by immersing them in ethanol solution and SEM (scanning electronmicroscope) evaluation confirmed the presence of the ZnO coating. The frictional forces were calculated between the wiresand brackets in four groups: group ZZ (coated wire and bracket), group OO (uncoated wire and bracket), group ZO (coatedwire and uncoated bracket) and group OZ (uncoated wire and coated bracket). Kolmogorov-Smirnov, Mann-Whitney andKruskal-Wallis tests were used for data analysis.

***Results.*** The frictional force in ZZ (3.07±0.4 N) was the highest (P <0.05), and OZ (2.18±0.5 N) had the lowest amount of friction (P <0.05) among the groups. There was no significant difference in frictional forces between the ZO and OO groups (2.65±0.2 and 2.70±0.2 N, respectively).

***Conclusion***. Coating of porcelain bracket surfaces with ZnO nanoparticles can decrease friction in the sliding technique,and wire coating combined with bracket coating is not recommended due to its effect on friction.

## Introduction

Tooth movement is an essential part of orthodontic treatment. Sliding the tooth on an orthodontic wire‏ is one of the techniques in this context, with advantages‏ including‏ a decrease in chair time, patient comfort and 3-dimensional control of tooth movements.^1^On the other hand, one of the major disadvantages of this technique is the wire-bracket friction, requiring application of higher forces to overcome it, which endangers the anchorage.^2^ In order to induce tooth movements, it is necessary to apply mechanical forces in the range of 100‒200 g on the tooth. Friction between the wire and bracket, which opposes tooth movement,‏ is associated with sliding movements. Subsequent to application of forces on the tooth, tipping movements begin, creating an angle between the bracket and the wire. When such an angle reaches a threshold, a contact is created between the wire and bracket margins, resulting in adhesion between metallic surfaces. Then, the wire gradually undergoes notching and plastic deformation. All these phenomena, in turn, prevent continuous tooth movements, leading to intermittent halts in tooth movement.^3^To overcome such a problem, the applied force should increase up to 40‒60%‏ of the initial force. On the other hand, any increase in the amount of applied force increases the risk of anchorage loss, which is considered an unfavorable‏ event‏ in orthodontic treatment. In addition, an increase in the amount of force increases the risk of root resorption.^4^

At present the use of ceramic brackets which are more esthetic than steel brackets is on the increase‏ but these brackets exhibit significantly higher frictional resistance compared to steel brackets.^5^ Differences in frictional resistance between‏ steel and ceramic‏ brackets are attributed to the surface characteristics of ceramic brackets.^6^Up to now, different techniques have been introduced to overcome such a problem, including the use of wires with different shapes and sizes or different chemical compositions and also the use of extraoral forces and temporary implants.^7^Use of nanoparticles with spherical structure was introduced in 1990s as solid lubricants. This technological achievement has been considered to decrease friction between metallic surfaces. Redlich et al^8^ evaluated the amount of decrease in frictional forces between stainless steel orthodontic brackets and wires after covering the wires with saturated nickel-phosphorus and tungsten disulfide nanoparticles. The results showed a significant decrease in frictional forces of the samples.‏ Samorodnitzky et al^9^ evaluated the effect of depositing spherical tungsten sulfide (WS_2_) nanoparticles on decreasing friction in nickel titanium (NiTi) orthodontic wires. The results showed that WS_2_-containing wires exhibited a significant decrease in friction in all the three tests; even AFM(atomic force microscope) analysis showed 4‒7 folds of decrease in frictional forces in wires containing WS_2_ compared to wires with no coating. In a study by Wei et al,^10^depositing a nano-layer of CNx (a carbon nitride film)‏ on the stainless steel wires resulted in a decrease in friction.

Goto et al^11^ showed that depositing ZnO particles on stainless steel substrate in vacuum decreased friction. Prasad et al^12^ reported that depositing WS_2_ particles on Iconel (a family of austenite nickel-chromium-based super alloys) substrate resulted in a decrease in friction and adding ZnO increased these effects several folds. Katz et al^13^ showed that WS_2_ nanoparticles significantly decreased friction in archwires. Obviously, future clinical use of coated wires, brackets or both of them will depend on using safe biocompatibility materials according to accepted procedures. In particular, zinc oxide has been used in many areas, such as catalysis,^14-16^ gas sensors,^17^ and recently as lubricants.^18-19^In our previous study we showed that ZnO nanoparticles coating of stainless steel archwires decreased friction during sliding within stainless steel brackets.^20^In another research, we tried to decrease friction between stainless steel wires and ceramic brackets by depositing ZnO nanoparticles on the wires but we found no significant differences between coated and uncoated wires.^21^To the best of our knowledge, this is the first timethe porcelain brackets are coated with ZnO nanoparticles for decreasing frictional forces during sliding mechanics. In line with our previous studies, the present study was carried out to investigate the effect of depositing spherical ZnO nanoparticles upon porcelain brackets and stainless steel orthodontic wires on frictional forces between the wires and brackets.

## Methods

This study did not involve the use of any animals or human data or tissues, and thus, an ethics approval was not required.

### Samples

This study included four groups, each group containing 30 samples: group ZZ (coated wire and bracket); group OO (uncoated wire and bracket); group ZO (coated wire and uncoated bracket); and group OZ (uncoated wire and coated bracket). The samples consisted of ceramic brackets of upper right central incisors of the standard edgewise 0.022-inch slot system (Orthotechnology, Florida, USA), and 0.019×0.025-inch rectangular SS wires in straight form (American Orthodontics, Washington, USA).The frictional forces between brackets and the wires were measured. The procedure explained below was used to deposit ZnO nanoparticles on the wires and brackets:

First the wires (or brackets) were immersed in ethanol for 30 minutes in an ultrasonic bath at 30°C and then transferred into a water bath at 80°C. The nanoparticles were completely dispersed within ethanol and then the wires (or brackets) were placed in this solution one by one. In addition, different duration times were evaluated in a pilot study to deposit the nanoparticles. SEM evaluations confirmed deposition of ZnO nanoparticles on the wires. Furthermore, SEM preliminary results showed that compared to other time intervals, the presence and distribution of nanoparticles were more satisfactory than when the immersion time was 30 minutes. Therefore, this technique was deemed appropriate for deposition.

### Testing

**Figure 1.**
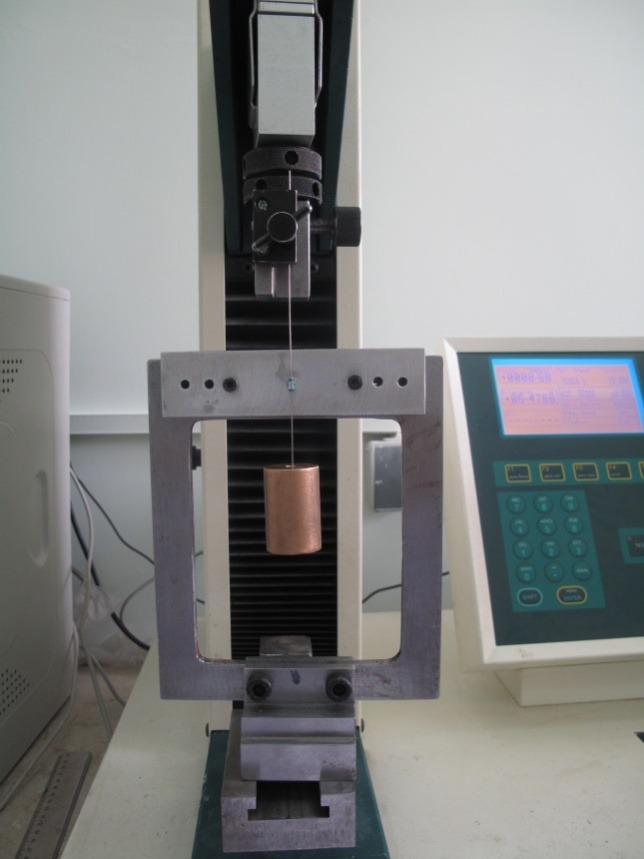
The method used to fix the bracket on the aluminum plate.

**Figure 2.**
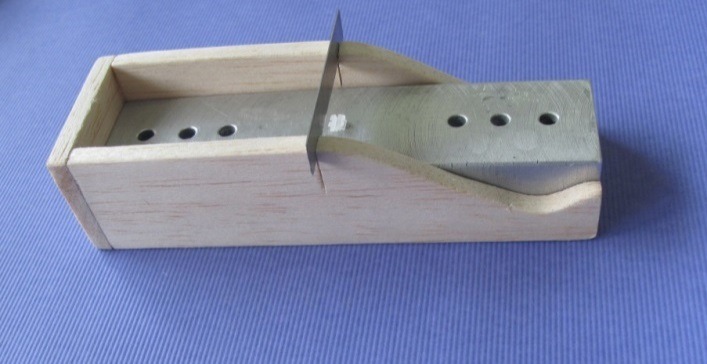
The universal testing machine used for force measurement.

After deposition of nanoparticles on the wires and brackets, the relevant tests were carried out to measure frictional forces during sliding. Therefore, the brackets were fixed on an aluminum plate using cyanoacrylate adhesive. To increase the accuracy of bonding and making sure that the bracket had been bonded with zero angle, a tool was designed to place the bracket and the aluminum plate at a fixed position of zero degree and to keep the position until complete setting of the cyanoacrylate adhesive (Figure 1).^21^A universal testing machine (Hounsfield Test Equipment: H5K Model, England) was used for pulling wires and creating sliding movements between the wires and brackets (Figure 2). The orthodontic wires were attached to the brackets using the elastomeric modules (Orthotechnology, Florida, USA). The upper end of the wire was attached to the upper arm of the universal testing machine and its lower end was attached to a 150-g sinker (Figure 2). The wire was pulled at rate of 0.5 mm/sec for 25 seconds and the force was measured using the universal testing machine.^21^To simulate the oral conditions, artificial saliva was poured on the bracket and wire every 3 seconds bya dropper.^7^ The bracket and wire were replaced after sliding of each sample so that the conditions would be similar for all the samples.

### Data analysis 

Kolmogorov-Smirnov test was used to evaluate normal distribution of data. Since the data was not distributed normally, Mann-Whitney and Kruskal-Wallis tests were used to evaluate differences between the groups. Data were analyzed using SPSS 16 and P < 0.05 was considered statistically significant.

## Results

**Figure 3.**
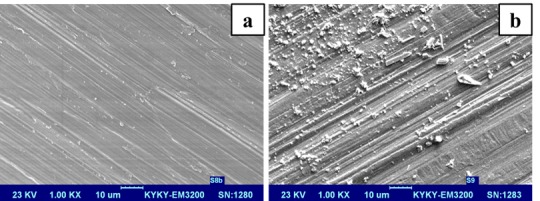
SEM images of the surface of the uncoated (a) and coated SS wires.

**Figure 4.**
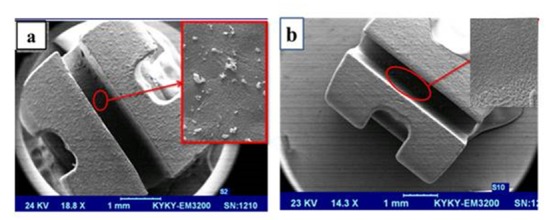
SEM images of the surface of the coated (a) and uncoated porcelain brackets.

The SEM images of the brackets and wires with and without deposition of ZnO nanoparticles are shown in Figures 3 and 4. In the OO group, the mean and standard deviation of frictional forces were 2.70±0.2 N, with 2.65±0.2, 2.18± 0.5 and 3.07± 0.4 N in the ZO, OZ and ZZ groups, respectively. The lowest frictional force was recorded in the OZ group (P <0.05), with the highest in the ZZ group (P <0.05). There were no significant differences in the means of frictional forces between the ZO and OO groups. The ZO group showed a decrease of 1% in friction compared to the OO group. OZ and ZZ groups showed a 17% decrease and a 13% increase in friction compared to the ZO and OO groups, respectively (Table 1).

**Table 1:** The results of descriptive analysis

**Group**	**Mean**	**95% confidence interval for mean**	**Median**	**Std. deviation**	**Minimum**	**Maximum**
ZO	2.65	2.53‒2.77	2.64	0.22	1.75	3.25
OO	2.70	2.56‒2.84	2.71	0.27	2.09	3.37
OZ	2.18	1.97‒2.39	2.18	0.55	0.71	3.25
ZZ	3.07	2.91‒3.22	3.14	0.41	>2.44	3.71

## Discussion

This study was carried out to evaluate the effect of depositing spherical ZnO nanoparticles on decreasing the frictional forces in sliding mechanics. To the best of our knowledge, the present study was the first study that evaluated the effect of ZnO nanoparticle coating on friction of porcelain brackets.

In the present study the mean of frictional forces was maximal in the the ZZ and minimum in the OZ groups, with no significant difference between the ZO and OO groups. Our data showed that the presence of ZnO nanoparticle coating on porcelain brackets was more effective than the coating on wire. This can be contributed to the surface properties of porcelain brackets.^6^Since the high surface roughness of porcelain brackets is an important factor in the determination of frictional forces, modification of such surface may have more potential to reduce the sliding friction.

In the ZO group, the surface of the porcelain bracket was still rough; therefore, covering the wire with nanoparticles did not result in major changes in frictional forces, consistent with our pervious study,^21^ where friction was not significantly different between ZO and OO groups. In that study, we deposited ZnO nanoparticles on SS wire and measured friction during sliding the wire within uncoated porcelain bracket; then we compared it with the control group. In that experiment, we did not obtain any reduction in fractional force by coating the SS wires; therefore, we designed this study to coat the porcelain brackets with nanoparticles in an attempt to find a solution to decrease friction between porcelain brackets and SS wires. In another study on SS brackets and wires, we found significant decreases in friction when wires were coated with ZnO nanoparticle.^20^In the present study, the same phenomenon was observed but the decrease was not significant. The discrepancy in the results might be attributed to the fact that none of the studies above evaluated the effect of depositing nanoparticles on friction in the porcelain substrate; therefore, the results cannot be compared simply. The important tribology principle can be mentioned that friction between two surfaces is a unique phenomenon and depends on the opposition of the two surfaces involved; therefore, we cannot overgeneralize the results of the coating of one surface or material to another one. As a result, it is always necessary to carry out experimental tribometery tests to evaluate the effect of different factors on friction. The other difference of the present study from our previous works is the use of artificial saliva to simulate the oral cavity conditions.

The mechanism through which the amount of frictional force between the wire and bracket decreases after deposition of nanoparticles was explained by Rapoport et al^22^and Cizaire et al.^23^In the first stage, when the wire and the bracket slot are parallel to each other, nanoparticles function as a spacer, decreasing the number of asperities in contact with each other; as a result, friction coefficient decreases. Concomitant with increasing the angle between the wire and the slot, the amount of force at slot margins increases, increasing frictional forces in wires without nanoparticle deposits. It appears that at this stage in wires covered with nanoparticles, some of these particles become flaked and the path of motion becomes slippery. The condensed nanoparticles are slowly disintegrated under the application of force and undergo free failure at interfacial areas. Also in cases in which the two surfaces are stainless steel without nanoparticle coating, the friction coefficient increases, with further increases over time. It appears that such changes take place through oxidation and adhesion between the abraded surfaces. Therefore, deposition of ZnO nanoparticles decreases friction as a mechanism protecting wires against oxidation of metallic surfaces.^24^When nano-coated surfaces are subjected to excessive forces at interfacial areas, sliding occurs at coated areas. As a result, the friction coefficient decreases. In the present study, the increase observed in frictional forces in the ZZ group might be attributed to a decrease in clearance between the wire and the slot.

Other materials have also been used in the interface in order to reduce friction, such as WS2, CNx or powdery or compressed (not spherical nanoparticles) ZnO. Prasad et al^25^ and Zabinski et al^26^ attributed a decrease in friction coefficients in ZnO deposits to their nanocrystal structure. On the other hand, ZnO in both powder form and compassed disk form cannot create a slippery surface; however, ZnO with a nanophase and structure can create a slippery surface, with a friction coefficient of approximately 0.2 which is lower than its predecessors.^26^WS_2_ nanoparticles with a spherical carbon structure have been used, which are different from the nanoparticles used in the present study, i.e. ZnO, although their effects on decreasing friction are similar. One of the advantages of ZnO in comparison to WS_2_ is the fact that it is biocompatible and safe for human health. Based on the reports available, 3T3 tests have not shown any toxicity of ZnO on human cells;^28^ however, more meticulous studies are needed to determine the long-term effects on various tissues.

In the present study, in addition to measuring the frictional forces between the bracket and wire, we used SEM technique to evaluate surfaces and pattern of deposition of ZnO nanoparticles. These images clearly showed the presence of ZnO spherical nanoparticles on the wire and also on the porcelain brackets.

In the present study, we coated the porcelain brackets with ZnO nanoparticles for the first time and found that deposition of such nanoparticles significantly decreased the frictional forces between the SS wire and porcelain brackets in the sliding technique. Although laboratory conditions cannot simulate intraoral conditions exactly, one should consider the positive effects of nanoparticles on decreasing friction in other orthodontic appliances like self-ligating brackets and Ni-Ti archwires. Decreasing treatment time and the risk of root resorption are potential advantages of decreasing friction between brackets and wires.

## Acknowledgments

The authors would like to thank Tabriz University of Medical Sciences for their financial support.

## Authors’ contributions

MK and AB were responsible for the concept and study design. AB performed study experiments and the analysis. MKh performed the literature review and revised the manuscript. BD critically revised the manuscript. All authors have read and approved the final manuscript.

## Funding

This study has been funded and supported by Tabriz University of Medical Sciences.

## Competing interests

The authors declare no competing interests with regards to the authorship and/or publication of this article.

## Ethics approval

Not applicable.

## References

[1] Bednar JR, Gruendeman GW, Sandrik JL (1991). A comparative study of frictional forces between orthodontic brackets and arch wires. Am J Orthod Dentofacial Orthop.

[2] Nikolai RJ (1982). Bioengineering Analysis of Orthodontic. 1st ed.

[3] Chen WX, Tu JP, Xu ZD, Tenne R, Rosenstveig R, Chen WL, Gan HY (2002). Wear and friction of Ni-P electroless composite coating including inorganic fullerene-WS_2_ nanoparticles. Adv Eng Mater.

[4] Angolkar PV, Kapila S, Duncanson MG Jr, Nanda RS (1990). Evaluation of friction between ceramic brackets and orthodontic wires of four alloys. Am J Orthod Dentofacial Orthop.

[5] Nanda R (1997). Biomechanics in Clinical Orthodontics.

[6] Omana HM, Moore RN, Bagby MD (1992). Frictional properties of metal and ceramic brackets. J Clin Orthod.

[7] Thorstenson GA, Kusy RP (2001). Resistance to sliding of self-ligating brackets versus conventional stainless steel twin brackets with second-order angulation in the dry and wet (saliva) states. Am J Ortod Dentofacial Orthop.

[8] Redlich M, Katz A, Rapoport L, Wagner HD, Feldman Y, Tenne R (2008). Improved orthodontic stainless steel wires coated with inorganic fullerene-like nanoparticles of WS_2_ impregnated in electroless nickel-phosphorous film. Dent Mater.

[9] Samorodnitzky Naveh GR, Redlich M, Rapaport L, Feldman Y, Tenne R (2009). Inorganic fuullerne – like tungsten disulfide nanocoating for friction reduction of nickel-titanium alloys. Nanomed.

[10] Wei S, Shao T, Ding P (2010). Study of CNx film on 316L stainless steel for orthodontic application. Diamond And Related Mater.

[11] Goto M, Kasahara A, Tosa M (2008). Reduction in frictional force of ZnO coatings in a vacuum. Jpn J Applied Phys.

[12] Prasad SV, McDevit NT, Zabinsky JS (2000). Tribology of tungstan disulfide-nanoccrystalline zinc oxide adaptive lubricant film from ambient to 500°C. Wear.

[13] Katz A, Redlich M, Rapoport L, Wagner HD, Tenne R (2006). Self-lubricating coatings containing fullerene-like WS2 nanoparticles for orthodontic wires and other possible medical applications. Tribol Lett.

[14] Khatamian M, Divband B, Jodaei A (2012). Degradation of 4-nitrophenol (4-NP) using ZnO nanoparticles supported on zeolites and modeling of experimental results by artificial neural networks. Mater Chem Phys.

[15] Khatamian M, Khandar AA, Divband B, Haghighi M, Ebrahimiasl S (2012). Heterogeneous Photocatalytic Degradation of 4-Nitrophenol in Aqueous Suspension by Ln (La^+3^, Nd^+3^ or Sm^+3^) doped ZnO Nanoparticles. J Molecul Catalysis A Chem.

[16] Divband B, Khatamian M, Kazemi Eslamian GR, Darbandi M (2013). Synthesis of Ag/ZnO nanostructures by different methods and investigation of their photocatalytic efficiency for 4-nitrophenol degradation. ApplSurfSci.

[17] Yoon DH, Yu JH, Choi GM (1998). CO gas sensing properties of ZnO–CuO composite. SensorActuator.

[18] Zabinski JS, Corneille J, Prasad SV (1997). Lubricious zinc oxide films: synthesis, characterization and tribological behaviour. J Mater Sci.

[19] Goto M, Kasahara A, Tosa M (2011). Low-friction coatings of zinc oxide synthesized by optimization of crystal preferred orientation. Tribol Lett.

[20] Kachoei M, Eskandarinejad F, Divband B, Khatamian M (2013). The effect of zinc oxide nanoparticles deposition for friction reduction on orthodontic wires. Dent Res J.

[21] Kachoei M, Behroozian A (2010). Comparison of friction between ceramic brackets and ZnO nanoparticle coated wires. Iran J orthod.

[22] Rapoport L, Leshchinsky V, Lapsker I, VolovikY VolovikY, Nepomnyashchy O, Lvovsky M (2003). Tribological properties of WS_2_ nanoparticles under mixed lubrication. Wear.

[23] Cizaire L, Vacher B, Le-Mogne T, Martin JM, Rapoport L, Margolin A (2002). Mechanisms of ultra-low friction by hollow inorganic fullerene-like MoS_2_ nanoparticles. Surf Coat Technol.

[24] Friedman H, Eidelman O, Feldman Y, Moshkovich A, Perfiliev V, Rapoport L (2007). Fabrication of self-lubricating cobalt coatings on metal surfaces. Nanotech.

[25] Prasad SV, Zabinski JS (1997). Tribological behavior of nanocrystalline zinc oxide films. Wear.

[26] Zabinski JS, Sanders JH, Nainaparampil J, Prasad SV (2000). Lubrication using a microstructurally engineered oxide: performance and mechanisms. Tribol Lett.

[27] Rakhshani AE, Kokaj J, Mathew J, Peradeep B (2007). Successive chemical solution deposition of ZnO films on flexible steel substrate: structure, photoluminescence and optical transitions. Appl Phys A.

[28] Schilling K, Bradford B, Castelli D, Dufour E, Frank Nash J, Pape W (2010). Human safety review of “nano” titanium dioxide and zinc oxide. Photochem Photobiol Sci.

